# Mycoponics: Controlled Bioproduction Utilizing Biophysical, Solid‐State, Liquid Nutrient Delivery

**DOI:** 10.1002/biot.70184

**Published:** 2026-02-06

**Authors:** D. Marshall Porterfield, Simone X. Moulton, Adriana K. Sanchez, Anna Sorg, Tayla Koenig, M. Shane Terrell, Sigrid Zahner, Alex Baena, Caitlin Proctor, Richard J. Barker

**Affiliations:** ^1^ Department of Agricultural and Biological Engineering Purdue University West Lafayette Indiana USA; ^2^ School of Aeronautics & Astronautics Purdue University West Lafayette Indiana USA; ^3^ Natural Resources and Environmental Science Program Purdue University West Lafayette Indiana USA; ^4^ Department of Art and Design Purdue University West Lafayette Indiana USA; ^5^ School of Sustainability Engineering and Environmental Engineering Purdue University West Lafayette Indiana USA

**Keywords:** bioproduction, mycelia, mycology, mycoponics

## Abstract

Mycoponic biotechnology, inspired by hydroponics—a vital technology for agriculture research and space exploration, is limited by innate substrate contamination commonplace in commercial mycoproduction. Using micro‐structured ceramic tubes as “substrate,” mycoponics provides mycelial cellular filaments with an air‐phase/solid‐state, antimicrobial interface for cellular liquid nutrient media uptake. We show how antimicrobial biophysical size exclusion facilitates mycoponic “persistent‐filtration‐defense” (PFD), experimentally confirmed using flow cytometry, and electron microscopic analysis of the interfacial ceramic pores (less than 300 nm). The antimicrobial mycoponic interface enabled development of a complete mycoponic nutrient medium, producing blue oyster mushrooms from liquid culture 2 weeks post inoculation. This completely eliminates grain‐spawn (2–4 weeks) and fruiting phase (2–4 weeks) times using granular substrate bags that require energy/time‐intensive antimicrobial processing. Mycelial colonization times decreased by 9 days, while biomass increased (170%) with activated carbon inside the tubes. Mycoponic cultivation of Reishi mycoleather gloves demonstrates direct 3D‐mycomaterials, and we show how mycoponics enables advanced scientific imaging (thermal) and techniques, including mycelial exudate recovery for drug discovery. Mycoponics enables hybrid solid‐state, liquid culture for continuous bioproduction of mycelial pharmaceuticals, representing 18% of the global market. The efficiency and extended cultivation enabled by mycoponics will facilitate significant future advances in mycology and mycoengineering.

## Graphical Abstract and Lay Summary

Mycoponic biotechnology, inspired by hydroponics—is enabled by antimicrobial ceramics as “substrate,” using a complete mycoponic media for continuous bioproduction of mycelial and mushroom products. Applications include food, mycoleather, and mycopharmaceuticals, representing 18% of the global market. The efficiency and extended cultivation time enabled by mycoponics facilitates future advances in mycology and mycoengineering.

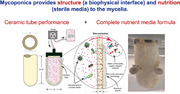

**Abbreviations**
MNM: mycoponic nutrient media

## 1 Introduction

### 1.1 Hydroponics: A Biotechnology and Tool of Science

Anthropological and agricultural origins of hydroponic technology include indigenous farmers from South America and China [1], where the use of ceramics in irrigation and hydroponics was first reported over 2000 years ago [2] in ancient Chinese text describing “buried clay pot irrigation,” which is still used in India, Pakistan, Iran, and Latin America [3]. In the 19th century, scientists had identified essential minerals [1], refined media formulas [1], and developed soil‐free horticulture methodology, with Stiles and Jørgensen [1] discovering the importance of rhizosphere aeration in solution. This set the stage for two parallel lines of progress in the 1920s–1930s: (1) rigorous scientific determination of plant nutrient requirements, led by Dennis R. Hoagland et al. [4, 5] at the U. of California, and (2) the visionary efforts to scale from the lab to the field, led by William F. Gericke (also at UC) who popularized and publicized hydroponics as a crop production technology [6].

Hoagland's enduring legacy is the comprehensive nutrient formula bearing his name. “The Water‐Culture Method for Growing Plants Without Soil” [7], published as a California Agricultural Experiment Station circular, which later became a seminal reference for researchers, codifying best practices, and canonizing “Hoagland's Solution” as the foundation for hydroponic media formulations. Hoagland and Arnon [7] also demonstrated the importance of aeration and pH for hydroponic optimization [4], establishing a baseline for consistent results, earning them the AAAS Newcomb Cleveland Prize in 1940 [8] for their work.

### 1.2 Hydroponics Goes to War: Scaling and Early Applications

Prior to World War 2, hydroponics was being scaled beyond the laboratory by Gericke [9], who coined the term in 1937 derived from Greek, “hydro” (water) and “ponos” (labor) [6], literally meaning “water‐working.” Working with Pan American Airways on Wake Island before WWII [6, 10], he demonstrated practical commercial productivity, prompting the US Army Air Forces (AAF) to develop hydroponics under the Quartermaster Corps [1, 11]. Hydroponics operated on Ascension Island (Atlantic), Iwo Jima and Hawaii (Pacific), Ponape in Micronesia, Atkinson Field in Guyana, and Wright‐Patterson AFB (Ohio). During the war, Army Air Corps Major Daniel Arnon, PhD, was stationed on Ponape Island (1943–46), overseeing a gravel‐based hydroponics operation [12], with his success mirroring other US Army hydroponics projects in demonstrating the strategic asset of biological in situ resource utilization (BISRU) in austere operating environments.

### 1.3 Hydroponics: To Boldly Grow Where no One Has Grown Before

In the 1950s–60s, hydroponics evolved from wartime experiment to commercial technology [11, 13] and, by the dawn of the Space Age, hydroponics had been incorporated into America's first lunar outpost plans, the US Army's Project Horizon (1959) which included “controlled ecological life support systems” (CELSS) with hydroponics to supply food/oxygen to lunar crews [11, 13, 14]. NASA and Air Force studies in the early 1960s advanced space farming and “biologistics in space [10]” demonstrating compact, closed‐loop, high‐yield crop production under electric light. These early studies drew on the US military's wartime experience, leveraging hydroponics for bioregenerative habitation for sustainable operations [11, 15]. Later in the 1980s, NASA established the CELSS program at Kennedy Space Center (KSC), leading to mature test beds for human‐rated trials [11, 14, 16] that were later canceled before implementation [16, 17]. Managing water/nutrients in microgravity is key for leveraging space hydroponics, where microgravity inhibits water drainage, promoting rhizosphere hypoxia [18].

The porous tube plant nutrient delivery system (PTPNDS) developed by NASA CELSS in the late 1980s [19, 20] uses semi‐permeable tubes (porous ceramic/hydrophilic membranes) supplied with hydroponic media [19, 21]. The primary engineering driver was controlling water in microgravity by maintaining variable vacuum (suction) inside the tube, drawing media through the tube, allowing interfacial roots access to water/nutrients while controlling water potential [21, 22]. The Astroculture experiment [23, 24] demonstrated PTPNDS performance during a 1996 Space Shuttle mission, examining seed germination and root growth [18, 22, 25]. Hydroponic crop production in CELSS architectures (food/oxygen) will require 93 kg/year/person of hydroponic minerals [26] regenerated from human/crop waste [27, 28]. Therefore, fungi will be invaluable in a spaceflight bioregenerative life support system (BLiSS), by recycling/upcycling nutrients and inedible biomass and producing valuable biocommodities [29], like they already do in our terrestrial ecosystems, generating food, materials, and 18% of global pharmaceuticals [30].

### 1.4 Mycoponics: Hydroponics for Mushrooms

Globally, production of domesticated mushrooms utilizes granular nutrient substrates (grain, straw, bark, wood chips), requiring energy‐intensive processing into solid bag systems [31] that may be unsuitable for spaceflight because of biophysical diffusion limitations in redox transport in microgravity [18, 22]. Mycoponic technology potentially solves the biophysical redox problem by eliminating both bags and substrates restricting redox exchange, but requires a complete mycoponic nutrient medium (MNM) comparable to Hoagland and Arnon's [7]. Early adaptations of hydroponics for mycocultivation have shown promise, but ultimately reinforce contamination as the primary technical challenge for mycoponic cultivation of fungal organisms.

Unlike plant hydroponics, contamination is exacerbated by the use of nutrient‐rich organic media. Aksu and Günay grew *Agaricus bisporus* and *Pleurotus ostreatus* using basic mycoponic approaches, soaking inert media (perlite and tuff) with simple glucose solutions [32], demonstrating feasibility but low yields. Bechara et al. developed mycoponics for *A. bisporus* using simple media and a modified nutrient film technique that demonstrated mycelial colonization but limited mushroom production compared to commercial‐scale [33]. These results highlight nutrient solution and environmental contamination in mycoponics. Inert ceramic beads have also been used as an artificial substrate with nutrient media to grow *Agrocybe cylindrica* and *P. ostreatus* [34]. Nutrient‐loaded inert ceramic substrate pellets in bag‐cultivation is an intermediate technology used commercially for *Trametes* polysaccharide K (PSK, Krestin), an important anti‐cancer pharmaceutical [35]. Contamination control is the universal problem in mycoculture, which is exacerbated by the requirement of enriched nutrient media for mycoponics.

## 2 Materials and Methods

### 2.1 Fungal Cell Culture

 All mycelial inoculations of mycoponics tubes were made from liquid cultures grown in basic cell culture media.  Preparation of our basic culture media includes 600 mL of dH_2_O heated to 80°C and stirred magnetically with 1.4 g malt extract, 1 g peptone, and 0.1 g gypsum, individually poured into the beaker. Then, using a scale, measuring scoop, and spoonula, weigh 18 g of corn syrup and pour it into the beaker. Add 2–3 drops of blue food dye, or until the liquid mixture is a light blue color. Mix and continuously heat the basic culture media until fully homogenized. All media is autoclaved and maintained in liquid cell culture using custom mason jars containing liquid culture media (Lids with ∼2 syringe ports, 0.22‐micron filter). Cell cultures are grown until needed for inoculating the mycoponics tubes.  We obtained dikaryotic liquid cell cultures from commercial sources to create our own primary liquid culture for mycoponic inoculations. Primary liquid cultures of *P. ostreatus*, strains Harbor Blue P01 and Snow‐White Oyster Po3, were purchased and shipped along with *Trametes versicolor*, cultivar Versicolor and *Ganoderma lucidum* from North Spore 921 Riverside Street, Portland, Maine 04103.

### 2.2 Mycoponics Media Formulation

 The development of specialized MNM formulations represents a critical advancement in enabling substrate‐independent mycelial growth and basidiocarp production in the mycoponics system. A progressive optimization approach was implemented to develop a complete MNM that is functionally equivalent to Hoagland and Armon's hydroponic solution for plants. This methodological refinement evolved through three distinct formulations, each addressing specific limitations observed in preceding iterations, while maintaining bioavailability of essential metabolic precursors as shown in Table 1. The media were all autoclaved before being introduced into the inoculated mycoponic tubes for growth experiments.

**TABLE 1:** Composition and performance characteristics of Mycoponics nutrient media (MNM) solution formulations.

Component	Formulation		
	v1	v2	v3
Water	600 mL	600 mL	1500 mL
Corn syrup	54.0 g (8.2%)	20.0 g (3.0%)	110.0 g (7.0%)
Malt extract	3.9 g (0.6%)	3.9 g (0.6%)	8.0 g (0.5%)
Peptone	3.0 g (0.5%)	3.0 g (0.5%)	7.0 g (0.4%)
Gypsum	0.25 g (0.04%)	0.25 g (0.04%)	0.7 g (0.04%)
Tryptic soy broth	—	3.0 g (0.5%)	7.0 g (0.04%)
Ammonium sulfate	—	—	0.5 g (0.3%)
Microcrystalline cellulose	—	20.0 g (0.3%)‐	5.0 g (3.0%)
Oak sawdust	—	—	5.0 g (3.0%)
Nutritional enzymes	—	—	2.0 g (1.0%)
Additional components	Food color	Blue oyster inoculum	—
Pretreatment	—	3 weeks at 25°C	Enzyme digestion^a^
**Growth characteristics**			
Mycelial growth	Sustained	Rapid/Good	Excellent
Fruiting body	None (6 months)	Partial, 2 months^b^	2 weeks/continuous
Exudates	High	High	Medium
Functional application	Inoculation culture	Exudate production	Complete life cycle

^a^ Enzyme digestion was performed for 24 h at 45°C with agitation (320 RPM).

^b^ Fruiting body formation was inconsistent after 2 months (18/6 light/dark).

Initial formulation development (v1) employed a predominantly simple‐sugar approach (8.2% corn syrup, 0.6% malt extract, 0.5% peptone, 0.042% gypsum) that successfully sustained robust mycelial colonization but failed to induce fruiting body formation despite extended cultivation periods exceeding 6 months (Figure 2). High production of exudates might reveal high metabolite production, suggesting active primary metabolism but insufficient activation of reproductive/developmental signaling, reinforcing established understanding that basidiomycete fruiting requires specific nutritional cues beyond those supporting vegetative growth. The second‐generation formulation (v2) reduced the corn syrup concentrations (3.0%) while incorporating microcrystalline cellulose (3.0%), which was digested with *Pleurotus* inoculum to provide a carbohydrate mixture more representative of natural substrates. The third‐generation formulation (v3) represented a shift to enzymatic pre‐processing of complex substrates. By incubating microcrystalline cellulose (5 g) and oak sawdust (5 g) with a consortium of hydrolytic enzymes (2.3 g) at 45°C for 24 h prior to incorporation with other media components and autoclaved. The resulting formulation consistently induced fruiting body formation while simultaneously reducing contamination susceptibility through decreased reliance on simple sugars. All media was made and autoclaved before being stored at 4°C before use on demand.

### 2.3 Mycoponic Slip Cast Molded Ceramic Fabrication

Slip mold cast ceramic fabrication is a well‐established technique to manufacture ceramics with complex shapes [36, 37, 38]. The process starts with preparing a ceramic slurry (slip) by dispersing ceramic powder in a liquid carrier with additives to control viscosity and flow. The slip is then poured into a porous mold, made of plaster, which absorbs water via capillary action, leaving a solidified layer on the mold walls. After sufficient time for forming the tube walls, the excess slip is poured out, and the molded tube is allowed to dry before firing [39]. Tube molds were constructed using 3D printed tube forms using the plaster casting technique to produce the 2‐part plaster molds used to reproduce the mycoponics tubes using slip cast ceramics as previously described for kaolinite water filters [40, 41, 42, 43]. A 3‐part plaster mold was fabricated, using a rubber glove as a form, for the 3D mycoleather gloves, which were assembled and clamped to seal the mold for slip casting.

We made two sets of forms, hands and tubes 5 cm in diameter and in varying lengths (10 cm, 30 cm, and 60 cm). The tube forms for making the molds were 3D printed to our specifications. The hand mold (plaster‐filled rubber glove) was divided into three unequal sections in order to prevent any undercuts when the mold was made. The tube mold was divided into two halves in order to prevent any undercuts when the mold was made. The exposed tube area was painted with three layers of green soap (surgery soap), which was allowed to dry in between each application. Again, clay walls touched the side of the tube at a 90° angle, and were erected around the section separately, contained by wooden bats, and then individually, poured with plaster, and this plaster was allowed to dry. The mold was then turned over onto the dried plaster base. The clay was carefully removed, the exposed tube repainted with surgery soap as before and divided into two equal half (quarter) sections with the clay walls as before, and plaster poured as before. This was repeated for both half (quarter) sections.

### 2.4 Slip Clay Formulas

**FIGURE 1:**
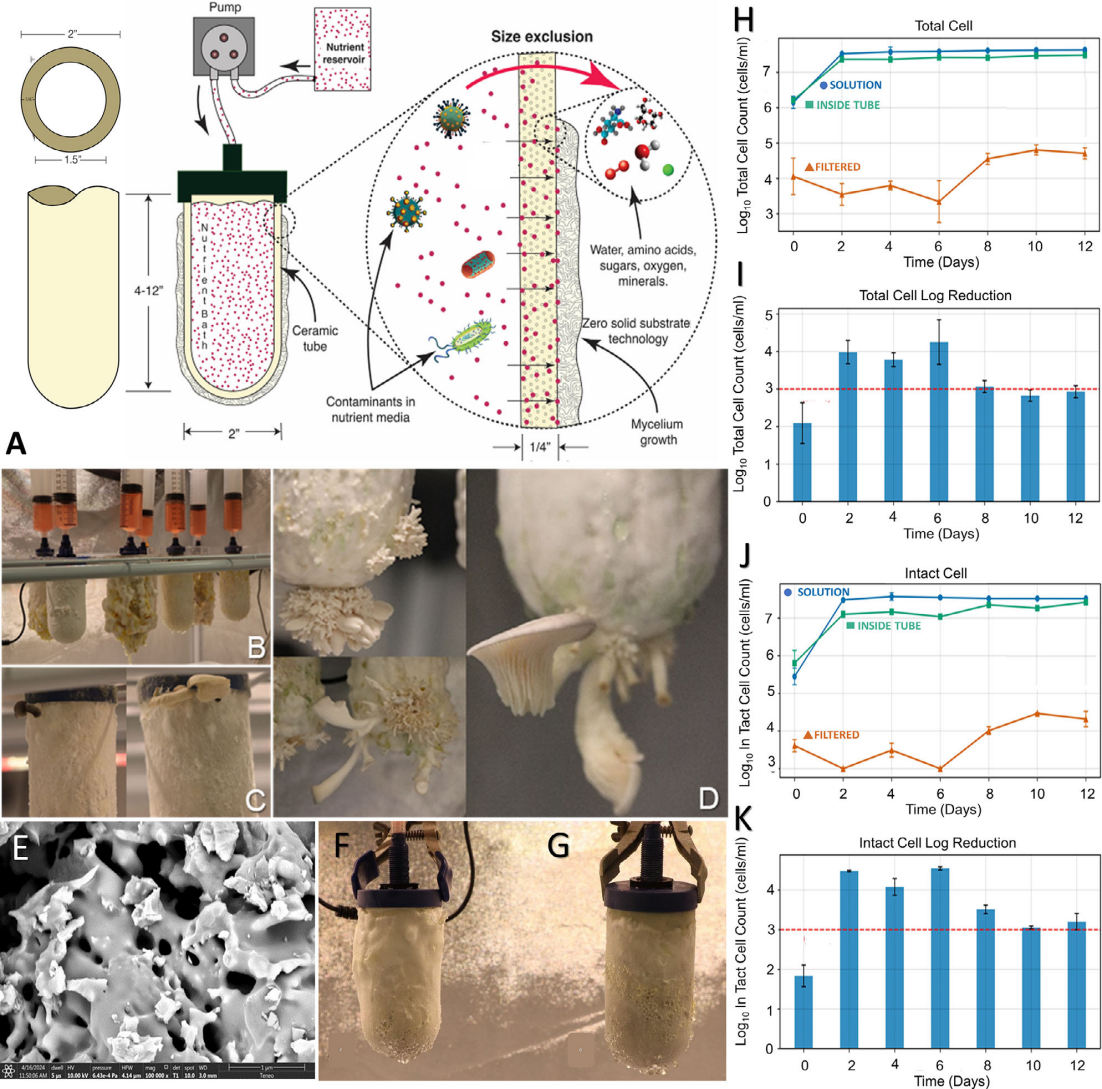
Schematic representation of the mycoponic technology showing the integration of porous ceramic technology with liquid nutrient delivery. The ceramic tubes were configured (A) with activated carbon (inner tube volume) to act as chemical and hydrological buffers. Nutrient delivery to the tube allows mycelial access to sterile media metered out through the cellular interface domain. This facilitates water, mineral nutrients, and catabolic substrate transport to the growing cells, in the interfacial domain between the atmosphere and the aqueous media, enhancing redox transfer and full aerobic productivity. Mycoponic performance was demonstrated by the growth and development (Movie S1) of mature basidiocarps (B–D) using the v3 MNM media formula (D), which includes the enzymatic conversion of lignocellulosic substrates (Table S1). Using the v3 MNM (Table S1) we demonstrated mushroom production within 2 weeks from inoculation (Movie S1) with liquid culture medium, eliminating grain spawn and solid substrates. We analyzed microbial contamination with ceramics, testing persistent filtration defense (PFD) using flow cytometry (H–K) to experimentally test size exclusion capabilities of mycoponic ceramics for whole (H, I) and intact cell (J, K) demonstrating size exclusion capabilities of the ceramic filter materials. scanning electron microscopy of the mycoponic cellular growth interface shows porous microstructures (E) down to 0.3 µm. MNM growth experiments with (F) and without autoclaving (G) validate the size exclusion performance of the mycoponic approach.

We mix our casting clay body in a specific order, larger to smaller particles into a mixture of the water into which soda ash has been fully dissolved. This is allowed to slake overnight (12–24 h) so that the particles can absorb all the water/soda ash mixture to saturation point. At this point we use a drill mixer to combine all the particles to a uniform consistency. The soda ash allows this to occur with minimum amount of water. Once uniformity is reached the sodium silicate is added to further deflocculate the casting slip to a specific gravity of approximately 1.65–1.8. Two Slip Formulas were used in these studies. For microbial water purification standard ceramic clay slip filters methods, using diatomaceous earth (DE) for water filtration were adapted. Mycoponic 10 cm mini‐tubes (Figure 1) are manufactured based on adapted methods [39, 42, 43, 44, 45] for creating antimicrobial ceramics using slip cast (mixed by adding in order and by volume, 29.36% water, 0.06% soda ash, 11.74% ball clay, 29.36% kaolin, 11.74% limestone, and 17.61% diatomaceous earth). This DE slip formula can be adapted by changing the content of carbon and DE in the ceramics mix for mycoponic tubes.

For the mycoleather hand, we used a “paper slip” formulation yielding an interface that demonstrated mycelial leather bioproduction with Reishi. This “paper clay” slip uses paper pulp content to modulate the porosity and pore size of the clay, instead of DE. The base clay slip by volume includes: 29.4% water, 29.4% Edgar plastic kaolin (EPK), 11.7% ball clay, 17.6% silica, 11.7% Custer feldspar, 0.06% soda ash, and 0.12% sodium silicate. This base slip is then mixed with paper pulp to make the final formula. EPK is an American‐sourced kaolin with excellent casting properties, used because of the large size of the kaolin particles, allowing for greater porosity. Ball clay (creates a more flexible body as the particles are the smallest), silica adds strength by vitrification of particles, and Custer feldspar (a high potash feldspar, 200‐325 mesh) helps lower the melting point of silica. Soda ash and sodium silicate can be substituted with the commercial deflocculant Darvan 7. The paper pulp is prepared in a separate bucket by shredding toilet paper to be mixed into the base clay at various percentages up to 20% by volume. We used a 20% volume paper pulp clay slip for the 3D mycoleather forms. Pulp preparation from toilet paper involves boiling, chopping and blending into a homogenous mix that is sieved of most water (30 mesh) before being added to the base paper slip. The pulp (20% volume) was added to the base slip and homogenized with a drill mixer. The 20% paper slip was poured carefully into the plaster molds as described above. After being poured, the clay tube was dried for 24 h in front of a fan. Before firing, imperfections were gently scraped off with a metal knife. The tubes are then fired at controlled temperatures. The hand forms were fired in oxidation at a bisque rate of firing (slow, 50°C per hour to 540°C to prevent abrupt quartz inversion/cracking) up to 940°C. The form was left to cool in the kiln until it reached room temperature.

### 2.5 Mycoponic Tube Assembly/Inoculation

To evaluate mycoponic PFD, we used commercial ceramics to fabricate mycoponic mini‐tubes using a 10 cm long × 5 cm diameter spherocylinder (a cylinder with a hemispherical end), commonly referred to as a “candle filter.” We evaluated commercial ceramic materials originally produced as ceramic water filters, with a porosity of 60% and an average pore‐size of less than 0.3 µm (verified using Scanning electron microscopy, SEM) in our flow cytometry studies. In all experiments, the tubes were filled (50% by volume) with granular (1–2 mm diameter) activated carbon (AC) (unless otherwise noted), and fitted with a 3D printed or form‐cast plastic cap with fluidic connectors. The cap and internal media can be modified depending on the fungal species being cultivated with different solid media and/or UVC sterilization to control internal microbial systems. UVC sterilization is also available to control microbial activity in the MNM media storage containers or fluidic delivery systems. Slip cast formulas can be adapted to achieve filtration control for human health standards and mycoponic performance.

The inoculation process starts with autoclaving the ceramic form (tube or glove) and handling it aseptically in a flow hood. Mature LC (2 weeks old) containing the fungi of choice is used for inoculating the dry tubes by pipetting sterile LC from the mason jars onto the bottom sphere of the ceramic form surface until it is saturated with mycelial liquid culture. This effectively creates an inoculation point at the bottom of the tube, saturated with liquid culture medium. The ceramic form is then internally filled with its first feeding of MNM. The tubes are then sealed in individual air‐tight containers and placed in a chamber at 20°C–25°C until the mycelia covers the ceramic interface in 2–6 weeks, depending on the tube size, mycelial inoculant concentration, strain/variety, and media formulation. After colonization is complete, the tubes can be moved into an open growth chamber with appropriate temperature, humidity, and redox controls.

### 2.6 Mycoponic Chamber Environment Conditions

After inoculation in controlled environment chambers, the tubes are moved into an open environment system and maintained at setpoint temperature/humidity and CO_2_ setpoints at 1000 ppm or less with HEPA filtration. Typically, the setpoint temperature for these experiments was 16°C, with humidity set at 85% and optimized for general mycelial growth with the high CO_2_ setpoint relative to that used for fruiting body production. The entire mycelial mass/surface is exposed to the gas phase environment, ensuring and facilitating optimal Redox exchange in the mycoponic mycelial environment. MNM delivery was done on schedule twice daily for all mycoponics MNM development experiments, whereas long‐term feeding was done on demand with fresh media re‐supplied to the syringes acting as individual mycoponic reservoirs (60 mL). Contamination was further limited by the use of peroxide and UVC light to decontaminate surfaces.

### 2.7 Flow Cytometry

Flow cytometry analysis of PFD was based on methods previously developed for water quality monitoring [41] conducted with a BD CSampler Plus using SYBR Green 1 and SYBR Green 1 plus propidium iodide dyes to find total cell count (TCC) and intact cell count (ICC), respectively (Franklin Lakes, New Jersey, USA). Samples were stained with dyes and incubated in the dark at 37°C for 13 min prior to analysis [41]. Filtered (cell‐free) Evian water was used to dilute samples as needed. Mycoponic tubes were configured for the analysis of the internal media relative to the inputs and to facilitate sampling relative to the surface media on the outside of the filter surface.

## 3 Results and Discussion

### 3.1 Porous Tube Ceramics for Mycoponic Nutrient Delivery Systems

Plant hydroponic technique theoretically offers significant and inherent advantages for fungi, but does not address contamination, a requirement to reliably produce mushrooms. Early mycoponic systems using inert substrates demonstrated viability but failed technically to match the minimal performance requirements for mycoponics, which include: (1) a biophysical interface for mycelia and mushroom growth, (2) a complete MNM supporting full fungal development, and (3) contamination control mechanisms. Unlike mycoponics, contamination control was not critical for PTPNDS, which use porous tubes (50% porosity, 0.5–5.0 µm pores) to provide mineral nutrients but lack size exclusion for microbial filtering.

For mycoponics, a “candle filter” delivery mode provides MNM (pump/syringe) into “closed‐ended” spherocylinder tubes (Figure 1A,B), as opposed to a recirculating reservoir design for PTPNDS [21]. Mycoponic fluidics are one‐way with uncirculated mycoponic nutrient media (MNM), prepared (autoclave or boiled) and dispensed on demand into the mycoponic ceramic tube (via syringe, pump, or gravity feed) into loose AC contained within the tube (Figure 1A–C). Ceramic fabrication utilizes a slip mold cast process [36, 38, 45] formulated to produce ceramic filters (porosity = 45%–55% and pore sizes of < 0.0.3 µm). These kiln‐fired ceramic tubes are functional as microbial filters, sterilizing the media as it is being filtered through the ceramics to the growing mycelium/filament. UVC sterilization can be applied to reservoir storage and fluidic delivery if needed. Here we report fundamental scientific and engineering principles for the design of mycoponic technologies based on (1) engineered ceramic substrates providing a biophysical mycelial interface for delivering (2) a biochemically complete MNM formula capable of supporting full fungal growth and development. We systematically tested the mycoponic ceramics while advancing MNM formulations.

### 3.2 Mycoponic Nutrient Media

Despite sterilization, contamination in conventional substrates (solid state fermentation) limits yield efficiency, consistency, and sustainability [31], because they remain highly susceptible to contamination during use due to their nutritionally rich nature and extended exposures [31]. A liquid MNM formula can avoid contamination through “on demand” sterilization from powdered/concentrated nutritional substrates like malt extract/tryptic soy broth. Industrial‐scale conversion of insoluble biomass, particularly agricultural waste, into biofuels is a critical research area [46], yielding new media and extending the mycoponics platform with industrially sourced nutrients. The mycoponic media evolved from simple (v1) to complex (v3) formulas using malt/TSB and enzyme digestion, producing mushrooms in 2 weeks (Figure 1, Movie S1) after transfer to mycoponic v3 MNM culture. This contrasted with inconsistent growth using the v2 MNM formula with *Pleurotus*, with high exudate production in v1/v2 formulas (Figure 1B,C), indicating active primary metabolism, but insufficient activation of reproductive basidiocarp development. This aligns with the understanding that basidiomycete fruiting requires specific nutritional cues beyond those supporting vegetative growth [31], with mycoponics providing the research platform to explore the nature of these cues.

The third‐generation formulation, v3 MNM, (Figure 1) represents a shift to enzymatic pre‐processing of complex substrates [47]. Microcrystalline cellulose (5 g) and oak sawdust (5 g) were incubated with a consortium of hydrolytic enzymes (2.3 g) at 45°C for 24 h prior to incorporation with other media [47] components to convert insoluble lignocellulosic materials into soluble oligosaccharides/monomers. This biomimetic engineering approach integrates natural fungal strategies of secreting extracellular enzymes [48] to digest complex substrates into absorbable nutrients, with the ceramic tube serving as a selective interface, analogous to the fungal cell wall. The v3 formulation consistently induces fruiting body formation within 2 weeks while simultaneously also potentially reducing contamination [48] by replacing bulk simple sugars supporting bacterial metabolism with complex carbohydrates (Figure 1D, Movie S1) that are inaccessible to the microbial enzymes.

### 3.3 Mycoponic Microbial Exclusion

Flow cytometry experiments confirmed performance [49] of the MNM used with mycoponics to sterilize the MNM by passive filtration through a ceramic interface (Figure 1E–K). We tested microbial filtration efficiency by measuring TCCs (Figure 1H,I) and intact cell (Figure 1J,K) counts of a controlled media sample to validate microbial filtration and exclusion via size‐based filtration by the ceramic microstructure. Scanning electron microscopy (Figure 1E) of the mycoponics tubes confirm the selective pore size is below 0.3 micron, allowing use of unsterilized media in experiments to evaluate mycoponic antimicrobial performance. Autoclaved mycoponics tubes (10 cm mini‐tubes with AC) were utilized in parallel cultivation trials with both autoclaved and non‐autoclaved nutrient solutions to assess the intrinsic contamination resistance capabilities of the system. As shown in Figure 1F,G, equivalent mycelial colonization patterns were observed in both the treatments with no detectable contamination in either scenario. The pore diameters were consistently maintained between 0.2 and 0.3 µm. This is significantly smaller than typical bacterial cells (0.5–5 µm) and fungal spores (3–30 µm), providing a physical exclusion barrier against potential contaminants. Flow cytometry analysis (Figure 1H–K) validated these observations, demonstrating filtration efficiencies exceeding 99.7%, with logarithmic reduction values matching standards for water filtration and confirming the size‐exclusion.

**FIGURE 2:**
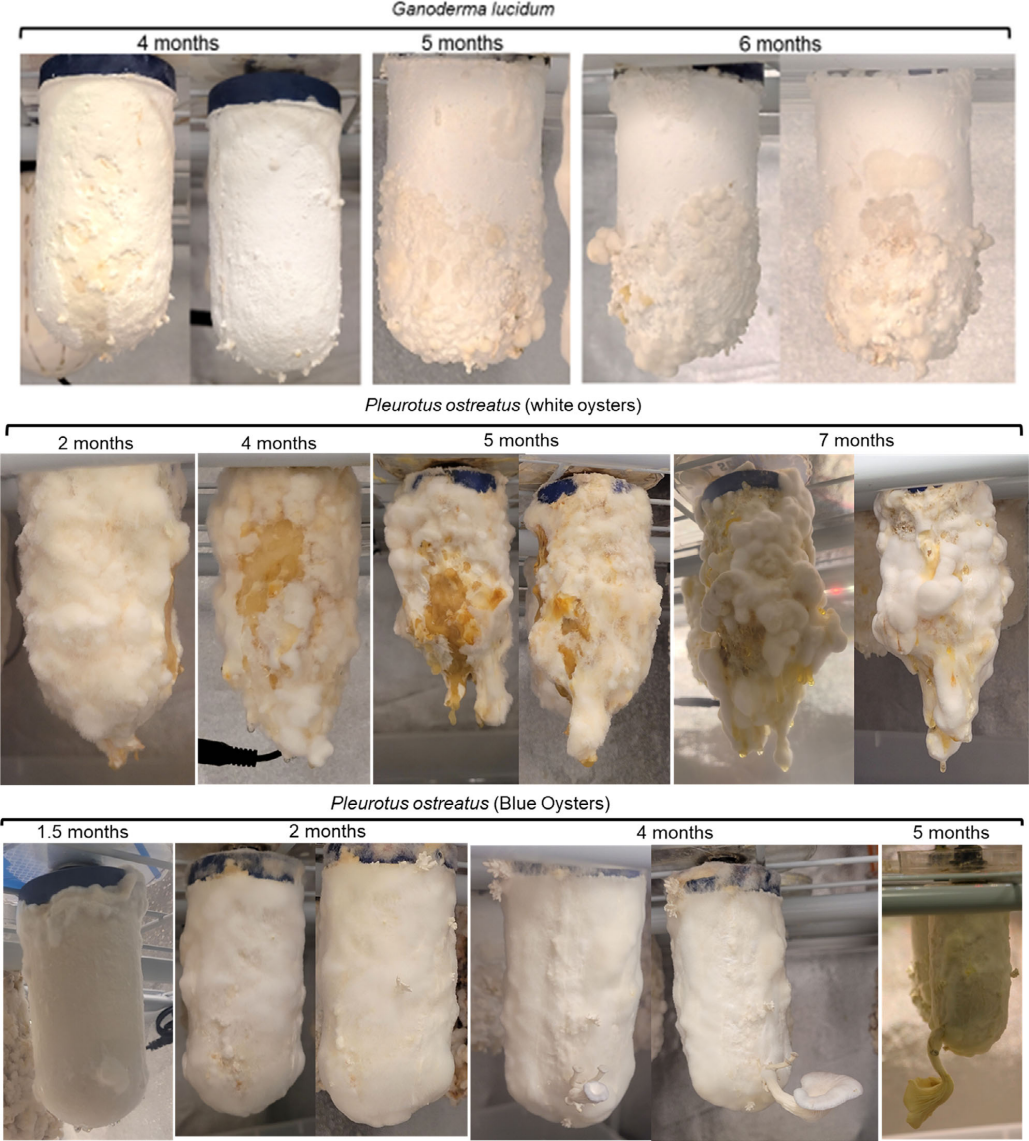
Comparisons of fungal and mycelial growth on 10 cm/4‐inch mycoponics “MiniTubes” grown using v1 (*Ganoderma lucidum* and *Pleurotus ostreatus* cv White) and v2 (*Pleurotus ostreatus* cv Blue) MNM with activated carbon. These were all grown under continuous illumination to promote fruiting but typically failed to produce primordia with simple media formulas (Table S1). Long term growth and development of the mycoponics demonstrates ceramic performance over time can outcompete simple bag substrates.

These findings demonstrate inherent mechanical mycocoponic decontamination (microbial filtration), reducing substrate processing energy costs [50] in commercial/pharmaceutical bioproduction. Size‐exclusion filtration is a mechanistically passive, but effectively “persistent filter defense” (PFD) physically addresses the fundamental challenge limiting conventional mycoproduction, microbial contamination. By eliminating solid substrate processing mycoponics PFD offers substantial energy savings and operational simplification. This intrinsic contamination resistance [45] enables extended cultivation cycles, where mycelial cultures maintained viable growth for over 7 months (Figure 2) without contamination, compared to typical 3‐month limitations of conventional bagged substrates [51]. These experiments establish pore size optimization for PFD as a critical parameter for substrate‐independent cultivation systems using porous materials (ceramics, plastics, metals, etc.). Size exclusion PFD between core and mycelial surfaces of mycoponic ceramics can maintain engineered microbiome communities inside the mycoponic tubes, as an approach to study/apply/engineer microbial/fungal associations in future bioreactors and bioprocess systems for circular biomanufacturing. Long term growth and performance of a variety of mycelial systems (Figure 2) demonstrate mycoponics can outperform bag substrates and show no signs of decreased performance of the MNM delivery after over 6 months of growth, more than doubling the average bag substrate system.

### 3.4 Mycoponic Materials and Performance

Using mycoponic PFD, it was possible to observe variability in mycelial morphology when comparing different and closely related species (Figure 2). Reishi grew robustly on tubes (Figure 2, top), which were harvested and tested as primary mycomaterials for mycoleather applications. The observed morphology was complex, suggesting the formation of fruiting structures. White oysters grew profusely over 7 months, assuming a “poodle” morphology and continuously producing copious amounts of thick liquid exudates (Figure 2, middle). Blue oyster culture [52] was the first to produce mushrooms with v2 media (Figure 2, bottom), with mycelia extending fractal structures at times, and reliably flushed to produce mushrooms with v.3 MNM (Figures 1 and 2 and Movie S1).

**FIGURE 3:**
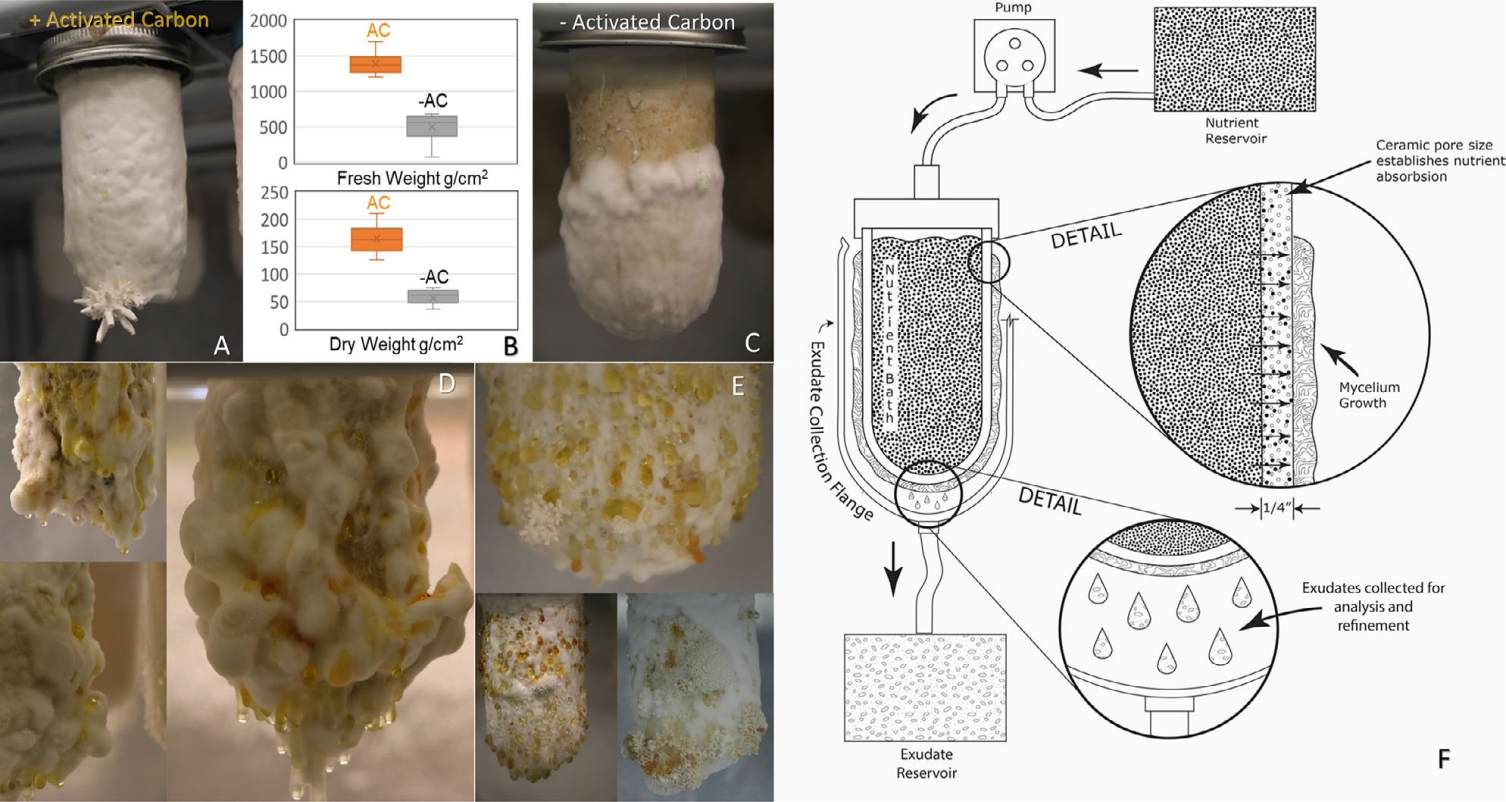
Mycoponic materials and mycelial exudate responses were evaluated with different MNM compositions and tube fillings, including studies evaluating activated carbon (AC) as a buffer and filler inside mycoponics. We compared AC filled tubes (A) to those without (C) and the growth responses (B) suggest hydrologic retention of the media in the capillary cohesive network of the carbon bed effectively optimizes MNM delivery and attenuates potential contamination by providing a highly favorable environment for the fungal mycelium both in terms of fresh (B, upper) and dry weight (B, lower), with statistical significance determined using the Student's *t*‐test (*α* = 0.05). Mycoponics is a platform to study and automatically induce mycelia and exudate production for pharmaceuticals, biochemical processing, to enable agro‐industrial applications in future green‐manufacturing and green‐chemistry platforms. Mycelial exudates are produced from white (D) and blue (E) varieties of *Pleurotus ostreatus* using mycoponics. White oysters prolifically produced exudates when grown in continuous light using v2 MNM. Blue oysters using v3 MNM were also highly productive in dark grown mycelia, with red‐colored exudates generating milliliter samples that could be recovered automatically (ongoing research) using a mycoponic flange (G) for passive/active recovery. These exudates represent a new class of bioactive sources for screening/discovering/manufacturing new antibiotics/ pharmaceuticals.

Our embodiment of mycoponics incorporates AC as an internal filling material, replacing soil humic acids [53] for “hydrological and chemical” buffering of the mycoponic system [54]. Mycoponic tubes with/without AC were inoculated with *P. ostreatus*, grown in darkness (6 weeks, v3 MNM), bioproduction assessed (fresh and dry weight measurements, growth rate calculation) with visual assessment of colonization patterns. The experimental results (Figure 3A–C) demonstrated enhancement of mycelial development, with AC increasing fresh (176.2%, *p* < 0.01) and dry (167.8% greater, *p* < 0.01) mycelial biomass when compared to unfilled tubes without AC (Figure 3B). Using AC, we observed denser, more uniform hyphae and colonization (AC maximum colonization −9.7 days).

AC enhances mycelial growth in mycoponics by: (1) increased surface area (500–1500 m^2^/g), providing adsorptive buffering of metabolites [55], where the microporous structures/gradients simulate “soil humic metabolism [56, 57]”; and (2) improvement of hydrological distribution of MNM gravitationally along the height (10 cm) of the tubes [58]. AC is a valuable optimization parameter for commercial mycoponics due to its simplicity, low cost, and biocompatibility. Future configurations of mycoponics will explore other filling materials for nutrient/pH buffering and controlled delivery and capabilities to integrate microbial bioprocessing as ecologically engineered symbionts with the mycelia maintained biophysically separated on the tube outside.

### 3.5 Mycoponics: Enabling Applications and Science

**FIGURE 4:**
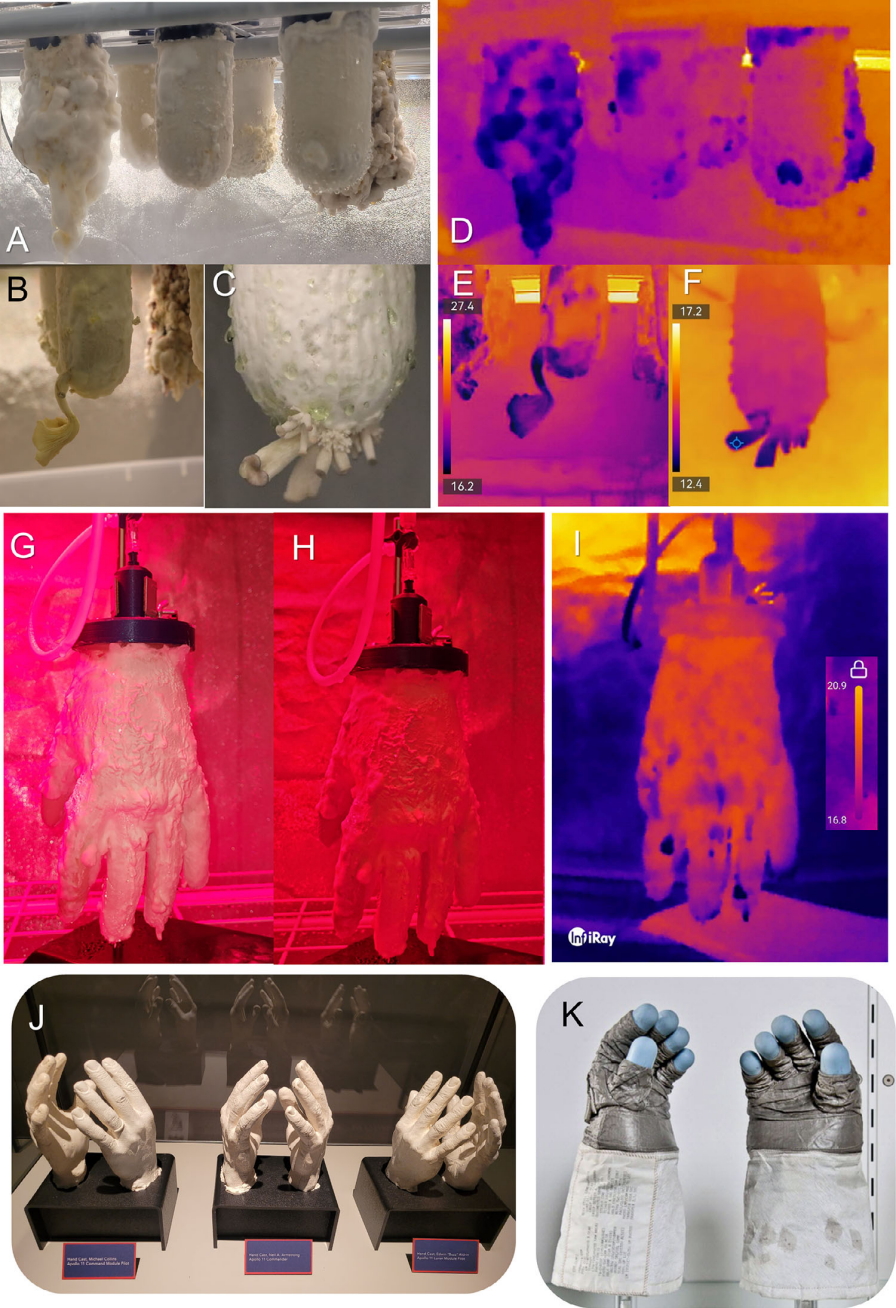
Optical and thermal imaging of mycelia and mushrooms growing on mycoponics reproduces previous reports [63] of fungal hypothermia and thermodynamic cooling during various phases of growth and development that are biophysically and morphologically important, but mechanistically not understood. Optical (A–C) and thermal images (D–F) can be compared to determine developmentally relevant metabolic patterns related to basidiocarp differentiation. The patterns of IR thermal (D–F, I) cooling have been physically confirmed using thermocouples (data not shown). This ongoing work is addressing fundamental questions concerning biophysical thermodynamics and mitochondrial respiratory waste heat [65] in chemoheterotrophs and cryophilic organisms. Mycoponics for growing 3D products like gloves (G–I) using *Ganoderma lucidum* have also been prototyped and tested with MNM v2, demonstrating 3‐D direct bioproduction of mycomaterials. We observed developmentally functional hypothermic patterns when growing mycoleather gloves (I) when imaged with IR thermography. Visible optical imaging of the mycoleather glove (G) can be compared to optical imaging (H) with externally applied IR (730/850 nm) for photobiomodulation [29, 66]. Astronaut crews require specialized and individually tailored equipment for exploration including gloves (J, K) bio‐manufacturable using 3D‐Mycoponics (G–I) to grow radio‐resistant mycoleather gloves derived from mycelial biomaterials. Panel J source, modified from "Plaster casts of astronaut hands" by Mary P Madigan, licensed under CC BY 2.0, and panel K is derived from "Expedition 50 Qualification Exams (NHQ201610250006)" by NASA HQ PHOTO is licensed under CC BY‐NC‐ND 2.0.

Mycoponics enables long‐term growth and study of fungal organisms (Figures 1, 2, 3) and experimentally observe the full ontology of these filamentous organisms in a scientifically transformative way. The early colonization period is susceptible to external environment contamination, but long‐term mycelial vigor overcomes most contamination. Long term cultures (Figures 1, 2, 3, 4) in standard growth chambers (80% relative humidity, 16°C, [CO_2_] ≤ 1000 PPM 100µM PAR) were viable and free of contamination for over 7 months. We observed differences in mycelial morphology in comparing long‐term growth of the entomopathogenic parasite Cordyceps grown using mycoponics (not shown), when compared to *Reishi*, and *Pleurotus* sp. (Figure 2), which correlate to patterned growth behaviors of the cellular filaments [29, 59]. In other experiments with Pleurotus, we could identify and recover mycelial exudates (Figures 2 and 3, red drops) by discrimination against MNM (dyed) to identify/characterize compounds produced by the mycelia (data not shown). A mycoponic flange is deployed to automate exudate recovery as a source of nutraceuticals, new drugs, and antibiotics (Figure 3F). Mycelially localized and extractable pharmaceuticals like PSK [35] can be harvested as a mycelial mass and regenerated for continuous bioproduction of mycelial‐based pharmaceuticals.

Mycoponics enables continuous solid state bioproduction of mycelial pharmaceuticals, offering global potential as both a tool for drug discovery and a scalable platform for continuous mycelial pharmaceutical bioproduction of fungal pharmaceuticals representing 18% of the global pharmaceutical market [30], including critical antifungals and antibiotics like penicillin, the world's first antibiotic discovered by Alexander Fleming in 1928 [60]. Penicillin, a byproduct of the *Penicillium rubens* mycelia [60], was discovered using Petri dish technology and revolutionized medicine before the widespread use of fungal antibiotics selected for bacterial antimicrobial resistance (AMR). There were 1.27 million deaths worldwide in 2019 from AMR [37], and methicillin‐resistant *Staphylococcus aureus* (MRSA) contributed to over 100,000 deaths [37]. New capabilities are needed to develop new antibiotics/treatments and mycoponic biotechnology is an enabling experimental platform to understand antibiotic and antimicrobial signaling [61] via controlled exposure of mycelia to biological targets and direct monitoring of fungal antimicrobial immunology [31, 61]. The fundamentals of fungal biology that have eluded science due to the mycelial lifestyle will now be accessible through mycoponics.

### 3.6 Fungal Hypothermia

Organismal biology approaches in mycology require isolation of the living thallus (collective filamentous body) away from any nutritive or physical substrate (Figures 1, 2, 3, 4). This is technically possible using mycoponics, providing unprecedented scientific access to all aspects of cellular physiology, biophysics, development, and reproduction. Because fungi grow distributed as cellular filaments within ecosystems, these organisms have not been readily observable [48, 62] or experimentally dissectible [62]. Fungal organisms are grown isolated as individual thalli using mycoponic PFD ceramics delivering MNM (Figure 4A–C). Thermal imaging (Figure 4D–F) revealed specific biothermodynamic growth patterns, confirming recent reports of fungal hypothermia [63]. Imaging (Figures 1, 2, 3, 4) and time lapse (Movie S1) of mycelial development reveals temporal and developmental patterns of mycelial biothermodynamics in thalli and basidiocarps of *Pleurotus*. Experimentally we maintained background (BG) conditions (16°C, 85% RH, [CO_2_] < 1000 ppm) to control water potential relative to transpirational flux. IR imaging reveals fungal hypothermia with dynamic developmental patterns observed in mycelia and fruiting bodies (Figure 4). Developmental morphology of vegetative and spore‐producing mycelia was compared (Figure 4A–C) to thermal images (Figure 4D–F), showing biophysical heterogeneity in fungal thalli related to growth activity. Meiotic sporogony is metabolically costly, localized within basidiocarps [64] (DNA, sporopollenin synthesis) physiologically specialized for spore dispersal [64]. Our experiments confirm that developmental patterns of fungal hypothermia are reproducible [63] using mycoponics observing whole individual mycelial organism.

## 4 Conclusions

Our work presents mycoponics as an enabling biotechnology for substrate‐independent mushroom/mycelial cultivation. The history of hydroponics includes NASA and microgravity‐based systems utilizing porous ceramics [24] for solid‐state crop production. Drawing from this foundation we developed mycoponics for bioproduction of fungal foods, biomaterials, and pharmaceuticals. Mycoponics enables long‐term mycological cultivation by addressing contamination, the primary factor limiting commercial production to short‐term, substrate‐dependent bag technology. Combining mycoponics contamination filtration porous ceramics with a complete MNM (v3), we have overcome contamination and nutritionally enabled long‐term bioproduction, while decreasing fruiting production down to 2 weeks using the complete V3 media developed using mycoponics. Overall performance was enhanced by incorporating AC within the ceramic tubes (Figure 1A), extending inherent contamination resistance and reducing cultivation cycles. Mycoponics enables non‐invasive experimentation, providing a transformative platform for drug discovery and bioproduction of mycelial pharmaceuticals, currently representing 18% of global products [30, 40]. Furthermore, mycoponics will impact the global mushroom market, with 48 million metric tons produced in 2021 and expanding at 7%–10% annually. Mycoponics will scientifically enable mycology and fungal biophysics, including phenomena such as fungal hypothermia [63]. Mycoponics is poised to revolutionize both fundamental mycological research and commercial industries.

## Author Contributions

Conceptualization: D. Marshall Porterfield. Methodology: D. Marshall Porterfield, M. Shane Terrell, Sigrid Zahner, Alex Baena, Tayla Koenig. Investigation: D. Marshall Porterfield, Simone X. Moulton, Adriana K. Sanchez, M. Shane Terrell, Caitlin Proctor, Alex Baena, Tayla Koenig, Anna Sorg. Visualization: D. Marshall Porterfield, Simone X. Moulton, Adriana K. Sanchez, M. Shane Terrell, Alex Baena. Project administration: D. Marshall Porterfield. Supervision: D. Marshall Porterfield, Sigrid Zahner, M. Shane Terrell, Caitlin Proctor. Writing – original draft: D. Marshall Porterfield, Simone X. Moulton, Alex Baena. Writing – review and editing: D. Marshall Porterfield, Simone X. Moulton, Adriana K. Sanchez, Sigrid Zahner, Caitlin Proctor, Tayla Koenig, Anna Sorg, Richard J. Barker.

## Conflicts of Interest

DMP is the primary inventor of the intellectual property claimed in this work and declares financial interests in mycoponic technology with four invention disclosures filed with Purdue University's Office of Technology Commercialization. There have been four total provisional patents filed, and two of these have been converted into full applications as of the date of publication. DMP is the majority owner of the startups Florence Quantum and MycoGravity LLC. MST is listed on a coinventor on two patent applications. SZ is listed on a coinventor on two patent applications. AB is listed as a coinventor on two patent applications and is the Owner/Science Officer for Monte Jorges Mycology Inc. SXM, AKS, CP, TK, AS, RJB declare no competing interests.

## Supporting information




**Supporting File**: biot70184‐sup‐0001‐SupMat.mp4.
Movie S1. mycoponic_mushroom_blue_oyster.mp4
Data available at: https://zenodo.org/records/16850510

## Data Availability

All data and materials used in the analysis in the main text or the supplementary materials are available from Zenodo. The dataset, identified by DOI https://doi.org/10.5281/zenodo.16877972, includes all raw and calculated data, including statistics. No specialized software is needed to access the data. The supplemental video is available also at Zenodo: https://doi.org/10.5281/zenodo.16850510.
